# Acute Aortic Dissection Masquerading as Testicular Torsion: A Case Report

**DOI:** 10.5811/cpcem.49096

**Published:** 2026-01-05

**Authors:** Bruce M. Lo, Megyn K. Christensen, Coral E. Byrns, Benjamin Chidester

**Affiliations:** * Sentara Norfolk General Hospital/Eastern Virginia Medical School at Old Dominion University, Department of Emergency Medicine, Norfolk, Virginia; † Eastern Virginia Medical School at Old Dominion University, Department of Emergency Medicine, Norfolk, Virginia

**Keywords:** aortic dissection, testicular torsion, case report

## Abstract

**Introduction:**

Aortic dissection is a rare but life-threatening condition with a high mortality rate if diagnosis is delayed. Aortic dissection classically presents with sudden-onset, sharp pain in the chest or back. However, atypical presentations can also occur, which could lead to a delay in diagnosis.

**Case Report:**

A patient initially presented to the emergency department (ED) with left testicular pain ongoing for several hours. On examination, he had tenderness in the left lower quadrant abdomen and left testicle. A testicular ultrasound revealed decreased blood flow to the left testicle, raising concern for testicular torsion. The patient was taken to the operating room, where no torsion was found, and he was subsequently discharged home. Several days later, the patient returned to the ED with worsening pain radiating to the back. A computed tomography revealed an acute type A aortic dissection extending to the iliac arteries. He was transferred for surgical repair and discharged 12 days later.

**Conclusion:**

While acute aortic dissection (AAD) typically presents with chest or back pain, atypical presentations can occur. When initial findings do not fully explain a patient’s symptoms, AAD should remain on the differential. This case highlights an uncommon presentation of AAD initially mimicking a testicular torsion.

## INTRODUCTION

Acute aortic dissection (AAD) is a rare but potentially life-threatening emergency. Timely diagnosis is critical, as delays are associated with significantly increased morbidity and mortality. Although AAD typically presents with sudden-onset chest or back pain, it can manifest with atypical symptoms that contribute to frequent misdiagnosis. While atypical presentations such as acute stroke or ST-segment elevation myocardial infarction have been documented, it is rare for a type A AAD to present initially as testicular pain. We present a case of a patient whose type A AAD was initially misdiagnosed as testicular torsion, resulting in a delay in diagnosis and definitive treatment.

## CASE REPORT

A 40-year-old male presented to the emergency department (ED) with sudden-onset left testicular and lower abdominal pain that began two hours prior to arrival. He denied any trauma or urinary symptoms, including hematuria or dysuria but reported pain and perceived swelling of the left testicle. On examination, the patient appeared uncomfortable, with tenderness in the left lower quadrant and left testicle, although no visible swelling was noted. He had no past medical history, nor did he have a family history of nephrolithiasis.

**Image 1:**
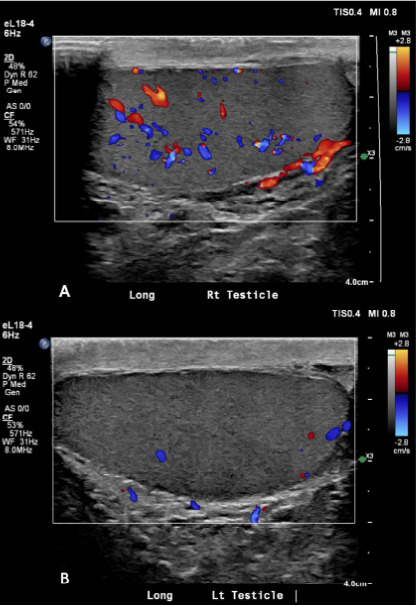
Testicular ultrasound with Doppler: A) right testicle showing normal flow; B) left testicle showing decreased blood flow concerning for torsion.

Initial vital signs were as follows: blood pressure, 101/58 millimeters of mercury (mm Hg); heart rate, 84 beats per minute (bpm); and temperature, 98.7 °F. Morphine was administered for pain control. Laboratory testing included a white blood cell (WBC) count of 13.2 kilograms per microliter (K/μL) (reference range 4.0–11.0 K/μl); hemoglobin (Hg), 12.8 grams per deciliter (g/dL) (11.7–16.1 g/dL); blood glucose, 114 milligrams (mg)/dL) (70–90 mg/dL); and creatinine, 1.3 mg/dL (0.8–1.4 mg/dL). A testicular ultrasound with Doppler demonstrated decreased blood flow to the left testicle, raising concern for testicular torsion (Image 1). A concurrent computed tomography (CT) of the abdomen and pelvis without contrast showed no renal stones but a calcification within the aorta. The patient was taken to the operating room for surgical exploration, but no torsion was found. He was subsequently discharged home following the procedure.

**Image 2:**
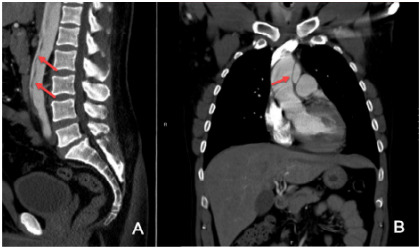
Computed tomography angiography of chest, abdomen, and pelvis: A) dissection flap down to iliacs (red arrows); B) dissection flap starting at the ascending aorta (red arrow).

Two days later, the patient returned with worsening left lower abdominal pain radiating to his back. He described the pain as sharp and tearing in nature. The patient denied any urinary complaints or drug use. Initial vital signs were as follows: blood pressure, 243/143 mm Hg; heart rate, 103 bpm; and temperature, 98.2 °F. Laboratory testing included a WBC count of 14.0 K/μL; Hg, 13.4 g/dL; blood glucose, 115 mg/dL, and creatinine, 1.1 mg/dL. A CT angiogram revealed a type A aortic dissection (AAD) extending from the distal ascending aorta to the iliac arteries (Image 2). The patient was then transferred for emergent surgical repair and was discharged 12 days later without complications.

*CPC-EM Capsule*

What do we already know about this clinical entity?

*Acute aortic dissection is rare, life-threatening, and often misdiagnosed due to highly variable and sometimes atypical presentations.*

What makes this presentation of disease reportable?

*This case presented with testicular pain with abnormal ultrasound findings suggestive of a testicular torsion, a rare presentation of type A aortic dissection.*

What is the major learning point?

*Unusual pain patterns or unexplained imaging should prompt consideration of aortic dissection in the differential, even in younger patients without risk factors.*

How might this improve emergency medicine practice?

*Maintaining suspicion for aortic dissection in atypical presentations can reduce misdiagnosis and improve patient outcomes.*

## DISCUSSION

Acute aortic dissection is a rare but life-threatening diagnosis that demands timely recognition and intervention. It is diagnosed in approximately one of every 12,000 ED visits and in one of every 1,000 patients who present with atraumatic chest pain.^1^ Approximately 67% of all dissections are classified as type A, involving the ascending aorta, while 33% are type B, involving a tear in the descending aorta.^2^ Data from the International Registry of Acute Aortic Dissection demonstrated that mortality for untreated type A dissections increases by 0.5% per hour, reaching 24% within 48 hours.^3^ The 30-day mortality rate for type A AAD is approximately 57%, whereas for uncomplicated type B dissections it is around 10%. However, the mortality for type B AAD increases to 25% when complications such as malperfusion or rupture are present.^2^,^4^

Chest pain is the most common presenting symptom, reported in 79% of patients with type A dissections.^2^ Although hypertension is a known risk factor for AAD, only 36% of patients with AAD present with elevated blood pressure at the time of diagnosis.^2^ The majority of patients are older, with an average age of 61 years.^2^ Diagnosis can be particularly challenging due to the wide variability in presentation. A systematic review analyzing 12 studies found that approximately 34% of patients with AAD were initially misdiagnosed.^5^ Common misdiagnoses included myocardial infarction, cerebrovascular accident, pulmonary embolism, musculoskeletal pain, ureterolithiasis, and psychological disorders.

The diagnosis in this case was particularly difficult due to several atypical features. The patient was significantly younger than the average AAD patient and had no known risk factors such as hypertension, illicit drug use, or signs of connective tissue disease such as Marfan syndrome. His presenting symptoms of left lower abdominal pain and testicular pain were also highly atypical for type A AAD, particularly in the absence of chest or upper back pain.

Only a few cases of aortic dissection presenting as testicular pain have been documented in the literature,^6^–^9^ and only one of these involved a type A dissection.^7^ In some of these cases, the theory as to why patients presented with testicular pain was secondary to expansion of the aorta leading to compression of the ilioinguinal or genitofemoral nerves.^7^, ^9^ To our knowledge, this is the first reported case in which a patient with type A AAD had an abnormal testicular ultrasound suggestive of testicular torsion suggesting a vascular involvement causing the initial pain. It is plausible that the dissection transiently compromised blood flow through the left testicular artery as it branches off the abdominal aorta, resulting in decreased perfusion on Doppler imaging and contributing to the initial misdiagnosis.

Computed tomography with intravenous contrast in arterial phase (angiography) remains the preferred imaging modality for diagnosis in the ED due to its rapid availability and high sensitivity. Other imaging modalities include point-of-care ultrasound, which can detect a dissection flap within an aorta but cannot rule out AAD. Additionally, clinical decision rules such as the Aortic Dissection Detection Risk Score along with D-dimer testing can assist in ruling out AAD in appropriately selected low-risk patients.^10^

## CONCLUSION

Acute aortic dissection is a rare but life-threatening diagnosis. Although patients typically present with chest pain or back pain, atypical presentations can also occur with AAD and should be considered, especially if the initial diagnostic workup is not negative.
